# Exploring the Roles of Trust, Attitudes, and Motivations in COVID-19 Decision-Making and Vaccination Likelihood: Insights from the Louisiana Community Engagement Alliance (LA-CEAL) Community—Academic—Public Health—Practice (CAPP) Partnership

**DOI:** 10.3390/ijerph22010048

**Published:** 2024-12-31

**Authors:** LaKeisha Williams, Leslie S. Craig, Erin Peacock, Tynesia Fields, Sara Al-Dahir, Frances Hawkins, Christopher Gillard, Brittany Singleton, Katherine Theall, Michelle Wilson, Gene D’Amour, Mai Vu, Christopher Sylvain, Lishunda Franklin, Kathryn Caldwell, Marie Krousel-Wood, Daniel Sarpong

**Affiliations:** 1 RCMI Center for Cancer and Health Disparities, Center for Minority Health and Health Disparities Research and Education, College of Pharmacy, Xavier University of Louisiana, New Orleans, LA 70125, USA; tfields2@tulane.edu (T.F.); saaldah@xula.edu (S.A.-D.); cgillard@xula.edu (C.G.); bsingle2@xula.edu (B.S.); gdamour@xula.edu (G.D.); daniel.sarpong@yale.edu (D.S.); 2 School of Medicine, Tulane University, New Orleans, LA 70112, USA; lcraig1@tulane.edu (L.S.C.); epeacoc@tulane.edu (E.P.); mwilso21@tulane.edu (M.W.); mawood@tulane.edu (M.K.-W.); 3 Department of Psychology, Tulane University, New Orleans, LA 70118, USA; 4 Baptist Community Ministries, New Orleans, LA 70130, USA; fhawkins@bcm.org; 5 School of Public Health and Tropical Medicine, Tulane University, New Orleans, LA 70112, USA; ktheall@tulane.edu; 6 St. Bernard Drugs, New Orleans, LA 70127, USA; mai.stbernarddrugs@gmail.com; 7 Best Life Pharmacy and Wellness, New Orleans, LA 70119, USA; csylvain@bestlifepharmacy.com; 8 Crescent City Pharmacy, Kenner, LA 70062, USA; crescentcityrx@gmail.com; 9 C & S Family Pharmacy, Metairie, LA 70005, USA; csfamilyrx@hotmail.com; 10 Office of Health Equity Research, School of Medicine, Yale University, New Haven, CT 06510, USA

**Keywords:** trust, COVID-19, vaccination likelihood, community—academic—public health—practice (CAPP) partnerships

## Abstract

Given the increasing integration of trusted leaders in public health science, including vaccination programs, context-specific understandings of community perceptions and levels of trust are critical to intervention. This study aimed to understand the perspectives and attitudes of the southeastern Louisiana community and inform the development of a community-engaged action plan. A cross-sectional survey of 555 southeastern Louisianans—including faith-based organization (FBO) members, community pharmacy (CommRx) customers, community-based organization (CBO) contacts, and Louisiana community-dwelling residents—was conducted from November 2020 to March 2021. Multivariable logistic regression was used to explore factors associated with COVID-19 vaccination likelihood. Of the participants, 89.9% were Black and 56.9% were women (mean age = 53.3 years). Doctors/healthcare providers (HCPs) were the most trusted COVID-19 information sources. Vaccination likelihood was associated with increasing age (odds ratio (OR) = 1.47; 95% confidence interval (CI): 1.27–1.72), trust in doctors/HCPs (OR = 2.83; 95% CI: 1.64–4.88), trust in government (OR = 4.26; 95% CI: 2.44–7.43), and motivations to keep one’s community safe (OR = 1.52; 95% CI: 1.36–1.70). CommRx customers (OR = 1.93; 95% CI: 1.02–3.65) and CBO contacts (OR = 2.57; 95% CI: 1.37–4.83) were more likely to receive a COVID-19 vaccine than FBO members. Engaging underserved communities and trusted stakeholders through collaborative Community–Academic—Public health—Practice (CAPP) partnerships such as the Louisiana Community Engagement Alliance can promote health and wellness and optimize health interventions.

## 1. Introduction

Widespread misinformation and disinformation during the COVID-19 pandemic highlighted the need for improved access to quality public health messaging delivered by trusted voices, particularly among underserved communities hardest hit by the disease [1,2]. COVID-19 vaccine acceptance varies globally and is influenced by several factors, including trust in healthcare providers and public health infrastructure [3]. Trusted messengers are individuals with longstanding, personal relationships and roots in communities, and often include personal healthcare providers (HCPs), pharmacists, and faith leaders, who can play a crucial role in increasing vaccine confidence [4,5]. However, despite being recognized as an integral link between social determinants of health (SDoH) and health outcomes due to their central role in promoting community health and wellness [5,6], trusted leaders often remain an ill-equipped and under-supported community resource for addressing vaccine hesitancy.

Recently, community–academic partnerships have gained traction as an opportunity to harness the potential of trusted community leaders [7,8,9,10]. Calls for meaningful partnerships between academic institutions, HCPs, and trusted community stakeholders have been issued as a means to reducing hesitancy, increasing vaccine confidence and acceptance, and building trust at all stages, from vaccine development to dissemination [10,11]. Yet, vaccine uptake remains context-specific, and variations in communities as well as socio-cultural determinants necessitate a nuanced approach to public health intervention, as demonstrated in previous studies that highlight how factors such as local beliefs, trust in government, and perceived vaccine safety significantly influenced COVID-19 vaccine decisions and acceptance/hesitancy [4,12,13]. Considering global trends in vaccine hesitancy and acceptance [3], the increasing integration of trusted leaders in public health science, including vaccination programs and health messaging, in addition to establishing a localized understanding of community perceptions and levels of trust, is critical to informing the evolving role of trusted leaders in their respective contexts [14].

The Louisiana Community Engagement Alliance (LA-CEAL) Community—Academic—Public health—Practice (CAPP) partnership was formed in November of 2020 to understand the disproportionate impact of COVID-19 on Black Louisianans and respond to community concerns about COVID-19 prevention and treatment strategies. Building on existing trusted partnerships with multiple stakeholders, including faith-based organizations (FBOs), community-based organizations (CBOs), federally qualified health centers (FQHCs), and community pharmacies (CommRx), the LA-CEAL CAPP partnership aimed to develop and execute a rapid community-engaged action plan to support COVID-19 vaccine acceptance among faith leaders, CBOs, and local pharmacists who were on the front lines in their communities, responding to the crisis and building awareness and public trust in the safety and efficacy of COVID-19 vaccines.

Via the LA-CEAL CAPP, we aimed to better appreciate the diverse perspectives and attitudes of the southeastern Louisiana community and inform the design and execution of a community-engaged action plan. To do so, this study examined trusted sources of information, COVID-19 preventive behavior engagement, motivations, likelihood of vaccination, and overall sentiment toward vaccination among a broad sample of southeastern Louisiana community groups, including FBO members, CommRx customers, CBOs contacts, and residents of Louisiana communities. Results and lessons learned regarding COVID-19 vaccination likelihood among patients receiving care in Louisiana FQHCs have been previously reported [15].

## 2. Methods

### 2.1. Target Population and Sampling

Given the disproportionate death and disease burden of COVID-19 among Black communities in Louisiana [16], the study was designed to oversample African American community residents. Using non-probability convenience sampling methods, 555 adult (i.e., ≥18 years of age) participants were recruited from CommRx customers, members of partner FBOs, individuals identified via CBO contacts, and residents of Louisiana neighborhoods. The analytic sample included 527 survey respondents with complete (i.e., non-missing) data on key variables of interest.

### 2.2. Survey Instrument and Data Collection

The survey was developed in coordination with national CEAL leadership and statewide CEAL team leaders via an iterative process that aimed to ensure the inclusion of key themes (e.g., knowledge, attitudes, beliefs, perceived risks and benefits, barriers and facilitators, trust in research(ers), SDoH, information seeking, and other behaviors) underlying COVID-19 research participation and the uptake of COVID-19 preventive strategies. Surveys were completed in person and at community sites, with direct data entry into study tablets or computers via the REDCap (Research Electronic Data Capture) platform. Trained study personnel and research assistants facilitated the self-administration or interviewer-assisted administration of surveys, according to participant preferences. All participants provided verbal informed consent. Study procedures were approved by the Tulane University Institutional Review Board and completed in accordance with institutional guidelines.

### 2.3. Study Measures

Socio-demographics (e.g., age, sex, race, education, income, and employment status) were self-reported. Age was estimated from the month and year of birth, with participants assigned a value of ‘1’ as the day of birth, and rescaled by dividing the original scale by 10 years. Participants were asked to self-report race using predefined categories (i.e., White, Black or African American, Asian, American Indian or Alaska Native, Native Hawaiian or other Pacific Islander, Other [please specify], or prefer not to answer). Those who selected “prefer not to answer” were classified as unknown. The “Other races” category included those who identified as “Asian”, “American Indian or Alaska Native”, “Native Hawaiian or other Pacific Islander”, “Other [please specify]”, and those who selected more than one race category. Educational attainment and employment status were collected via a single question. Annual household income was categorized as <USD 25,000, ≥USD 25,000, and unknown (i.e., for those who preferred not to answer this question).

Healthcare access and health literacy were also captured. Healthcare access was defined as having seen an HCP in the past 12 months, while low health literacy was defined as needing someone to help you read written information from your doctor or drug store, “sometimes”, “often”, or “always”. COVID-19-related SDoH were measured using questions asking about challenges faced by participants due to COVID-19 in obtaining needed healthcare (including mental health), having a place to live, having enough food to eat, having clean water to drink, obtaining needed medicine, and getting to where they needed to go. Each challenge was rated as “No, this is not a challenge”, “Yes, this is a minor challenge”, or “Yes, this is a major challenge”. Responses were dichotomized as major/minor challenge vs. not a challenge and summed to indicate the number of challenges experienced by participants (range 0–6).

To assess attitudes, motivations, and trust in COVID-19 messaging and behavioral recommendations, additional questions explored engagement in COVID-19 preventive behaviors, motivations to vaccinate, and trusted sources of COVID-19 information. Three preventive behaviors (i.e., wearing a face covering or mask, washing hands with soap or using hand sanitizer several times daily, and staying at least 6 feet away from others) were examined by asking about engagement in each practice in the past seven days. Engagement in preventive behaviors was positively coded if participants noted engaging in each of the three practices “very often” or “all of the time”. Reasons to receive the COVID-19 vaccine were grouped together, and summed scores were used to describe participant motivations to receive the COVID-19 vaccine. Those motivated by a concern for community safety and return to normalcy included respondents who answered yes to any of 6 items concerning wanting to keep family safe, wanting to keep community safe, wanting to keep one’s self safe, wanting to feel safe around others, not wanting to get really sick from COVID-19, and a belief that life will not go back to normal until most people receive a COVID-19 vaccine (Cronbach’s alpha = 0.9). Those motivated by health concerns/HCP’s advice included respondents who answered yes to having chronic health problems (e.g., asthma, diabetes) or having received a doctor’s recommendation to receive a COVID-19 vaccine (Cronbach’s alpha = 0.6). Higher scores indicated stronger motivation. Finally, trusted sources of information about COVID-19 included doctors/HCPs, faith leaders, family/close friends, work-/classmates, traditional news media, social media contacts, the U.S. government, the U.S. Coronavirus Task Force, the Louisiana state government, and local city/town government. Each item was measured on a 3-point Likert scale and dichotomized as “a great deal” versus “a little”, “not at all”, or “not applicable”.

The primary outcome measure was captured using a 7-point scale ranging from 1 (not at all likely) to 7 (very likely) in response to the question “How likely are you to get an approved COVID-19 vaccine when it becomes available?”

### 2.4. Statistical Plan

Descriptive statistics were used to describe the characteristics of the study sample. Means and standard deviations were computed for the continuous measures and frequency distributions obtained for categorical measures. The net promoter score (NPS)—a commonly used indicator of an individual’s experience that examines likelihood of service recommendation [17,18]—was derived using the 7-point Likert scale to gauge the likelihood of getting vaccinated. The NPS was calculated by subtracting the percentage with a score of 6 or 7 (considered “promoters”) from the percentage with scores between 1 and 3 (considered “detractors”). Those with a score of 4 or 5 were considered “passives” and were not included in the NPS calculation. NPSs range from −100 to +100. A score of −100 means all respondents are “detractors”, while a score of +100 indicates that every respondent is a “promoter”. The final outcome assessed likelihood of vaccination and was defined as a binary measure based on those with an NPS of 6 or 7. Stepwise logistic regression with backward elimination was performed to determine factors associated with vaccination likelihood, with a significance level of 0.10 set for removal from the model. All analyses were performed using Stata/SE 15.1.

## 3. Results

### 3.1. Sample Characteristics

The sample distribution included 47.3% FBO members, 19.2% CommRx customers, 15.8% Louisiana neighborhood residents, and 17.8% CBO contacts. Participants were predominantly of Black race (89.9%) with a mean age of 53.3 years (Table 1). The majority of the sample had a high school education or less (60.3%), and just over a third (34.5%) were employed either part-time or full-time. Nearly half of the sample (48.6%) reported an annual household income of less than USD 25,000. Regarding SDoH, 87.9% had seen an HCP in the past 12 months and few (14.2%) had low health literacy. Half of the sample (49.7%) reported not having any COVID-19-related challenges; among those affected by COVID-19 SDoH, obtaining needed healthcare (30.9%), getting where they needed to go (26.2%) and having enough food to eat (26.0%) were most often reported.

There were statistically significant differences across the four sampling groups in all characteristics except age, insurance ownership, and mean number of COVID-19-related challenges. Compared to FBO members, CBO contacts were less likely to be women (59.4% vs. 40.4%, *p* = 0.001). There were significantly fewer respondents of Black race among the neighborhood residents compared to the FBO member subsample (74.7% vs. 92.4%, *p* < 0.001). A greater proportion of CBO contacts reported an annual household income of less than USD 25,000 compared to their FBO counterparts (73.4% vs. 37.8%, *p* < 0.001). Low health literacy was more common among CommRx clients (26.7%) and CBO contacts (19.2%) compared to FBO members (8.8%, *p* < 0.001).

### 3.2. Attitudes Towards COVID-19 Messaging and Trusted Sources of Information

Compliance with each of the recommended COVID-19 preventive measures (face covering, hand washing, and social distancing) was high among sample respondents, ranging from 91.8 to 97.0%. Engagement in all three preventive behaviors was significantly lower among neighborhood residents compared with FBO members (75.9% vs. 89.6%, *p* = 0.007).

**Figure 1:**
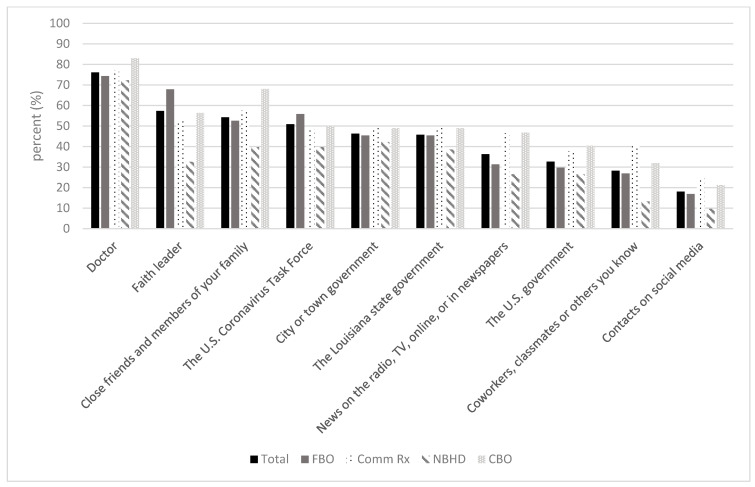
Trusted sources of COVID-19 information.

Across all groups, doctors/HCPs were the most trusted sources of information on COVID-19 (Figure 1). Other top trusted sources of information included faith leaders, family/close friends, and the U.S. Coronavirus Task Force. Social media contacts were least trusted across all groups.

### 3.3. Likelihood of COVID-19 Vaccination

Regarding likelihood of vaccination, the sample included 60.3% “promoters” compared to 25.8% “detractors”, giving a positive NPS of 34.5% and suggesting that respondents were likely to receive the COVID-19 vaccine when available (Table 2). Notably, the NPS varied across groups and was highest among the CommRx subsample (NPS = 44.6%), followed by the FBO (NPS = 33.7%), CBO (NPS = 31.9%), and neighborhood (NPS = 27.7%) subgroups.

Increasing age (odds ratio (OR) = 1.47; 95% confidence interval (CI): 1.27–1.72), trust in doctors/HCPs (OR = 2.83; 95% CI: 1.64–4.88), trust in government (OR = 4.26; 95% CI: 2.44–7.43), and the motivation to keep one’s community safe (OR = 1.52; 95% CI: 1.36–1.70) had higher odds of likelihood of vaccination (Table 3). In addition, CommRx customers (OR = 1.93; 95% CI: 1.02–3.65) and CBO contacts (OR = 2.57; 95% CI: 1.37–4.83) had significantly higher odds of likelihood of receiving a COVID-19 vaccine when available compared to FBO members.

## 4. Discussion

The analysis of participant data revealed varied perceptions of trusted messaging, vaccination motivations, and vaccination likelihoods among the four sampling groups. Likelihood of vaccination was dependent upon age, sampling group, trust in sources of information, and a general concern for community wellbeing, safety, and return to normalcy. These findings resonate with published evidence that vaccine likelihood at the outset of a pandemic is multifaceted and context-dependent, including correlations to SDoH and related inequities, structural racism, mistrust, and exposure to (mis)information [2,4,13,19]. In our sample, the strongest associations with vaccination likelihood were having a great deal of trust in one’s doctor/HCP, having a great deal of trust in the U.S. government, and being a CommRx customer or CBO contact (compared to an FBO member).

Trust, particularly trust in government vaccine approval/development processes, is a key factor underlying vaccine hesitancy [20]. Notably, despite the legacy of mistrust of government, research, and healthcare institutions among underserved communities [21], some studies have noted that Black adults are more likely than non-Hispanic White and Hispanic/Latina/o/x adults to consider federal agencies (i.e., CDC, FDA), local or state governments (e.g., departments of health), and/or public health officials credible or trustworthy information sources [4]. In our study, despite the U.S. government being ranked among the lowest trusted sources of COVID-19 information (seventh out of ten listed sources), high trust in the government was associated with being very likely to get vaccinated. Research among FQHC patients in Louisiana similarly revealed trust in doctors/HCPs and government sources of COVID-19 information to have the strongest and most consistent associations with COVID-19 vaccination likelihood [15]. These findings support the need for honest, consistent, high-quality public health messaging to build trust and confidence in vaccines. The early politicization of the pandemic [22,23], coupled with an onslaught of misinformation, disinformation, anti-vax groups, and conspiracy theories [1,13,24], likely fueled vaccine hesitancy and skepticism—a special concern and public health priority among communities with low institutional trust and low vaccination coverage rates [5]. Our findings align with the potential utility of a collective, robust communication infrastructure and multimodal approach using trusted, informed community messengers (e.g., HCPs, non-governmental entities, influential public figures, and religious communities) and different communication channels (e.g., community outreach and forums, traditional media, electronic outreach, direct conversations) to address reluctance and build confidence in vaccines [1,4,5,25].

Top trusted sources of COVID-19 information among the study population were doctors/HCPs, faith leaders, close friends/family members, and the U.S. Coronavirus Task Force, respectively. A recent systematic review similarly noted personal physicians as the most often mentioned type of trusted messenger, followed by one’s social network (e.g., friends, family, peers, neighbors), church and faith leaders, and local and/or state government officials [4]. The review further described other healthcare professionals (e.g., pharmacists), community leaders, and CBOs as particularly credible and trustworthy information sources among African Americans, with the added benefit of providing access to care and vaccine services [4]. Indeed, CBOs and FBOs were informal COVID-19 information dissemination sites in Louisiana throughout the pandemic, and community members would initiate conversations with trusted community voices and continue to seek reinforcement of this messaging from HCPs. Study findings thus further reinforce the need for a collective response to vaccine hesitancy, illustrating how healthcare professionals (e.g., doctors, nurse practitioners, pharmacists), FBOs, and CBOs, working in collaboration with science-based entities and connected to needed public health and clinical resources, can be a positive conduit for improved public health. Local CBOs understand the social and cultural dynamics essential to building mutually beneficial opportunities for community participation and outreach [2,9]. Ministries and FBOs are spiritual community assets that can help to meet community-wide needs while shepherding local communities in a health-promotion direction [26,27,28]. Doctors/HCPs are among the top trusted sources in their communities for health information and care [4], while pharmacists, given their strategic locations and flexible hours, are uniquely positioned for community response and engagement [5]. Ultimately, the LA-CEAL CAPP provided a collaborative entry point for all stakeholders to access vulnerable populations and share in the communication of COVID-19 information in ways most relevant to their own mission and most helpful to the missions of other collaborating groups (e.g., government, clinics, physicians, and community pharmacies).

Yet, beyond the trust and compliance of the general population, the success of public health interventions is reliant on multiple factors, including the effectiveness of communication methods and features of messaging, such as the content, style, and framing [4,25]. Possibly, due to messaging from their most trusted sources, more than 90% of surveyed participants were practicing COVID-19 prevention strategies. In addition, concern for community safety and return to normalcy were among the strongest factors associated with the likelihood of COVID-19 vaccination. These study findings suggest a sense of community and responsibility, and may also reflect a value for positive, hopeful messaging, as opposed to fear-based appeals, among this population. Indeed, evidence suggests that persuasive messaging using community-focused or collective action appeals (i.e., about protecting family members, including children and the elderly, as well as the “social other”) may resonate more strongly among African American communities than individual (i.e., self-focused) messaging to elicit a sense of unity and changes in behavior [4].

### Strengths and Limitations

These results should be considered in the context of the study’s limitations, including the unprecedented nature of the COVID-19 pandemic and the timing of the study. The study was launched in an effort to understand and respond to a trend of overwhelming devastation and potential vaccine uptake disparity among vulnerable communities and Black Louisianans. A significant strength of the study was the formation of LA-CEAL, and the collective impact of the community, academic, public health, and clinical practice entities involved. However, this study began early in the pandemic during the initial roll out of the COVID-19 vaccines, following months of exposure to COVID-19 messaging and the likely formation and setting of community members’ opinions. As a result, community perceptions, behaviors, and trust in health interventions may have shifted over time, and not be fully captured in this study’s findings. This study affirmed that community members rely on their FBOs, CBOs, CommRx, and HCPs to access healthcare and treatment options, and to understand the pandemic’s ever-changing messaging. In turn, the results also showed that social factors such as age, experience with messenger sources, and trust may influence behaviors, engagement, and decision-making. However, this study did not fully account for the influence of varying and sometimes conflicting media messaging on community beliefs and attitudes toward COVID-19. A deeper analysis of the media’s role in shaping community perceptions could provide insights into how different information sources influenced behavior during this period. Yet, the convenience sample reflects the perspectives, attitudes, and behaviors of a predominantly Black, adult population in southeast Louisiana, and the results may not be generalizable to community contexts outside of this race and region.

## 5. Conclusions

COVID-19 challenged the traditional silos of systems and institutions in the U.S., necessitating collaboration and the removal of barriers for the sake of community health, safety, and prosperity. The LA-CEAL CAPP partnership worked together to address COVID-19 vaccine-related disparities within vulnerable Louisiana communities, specifically among Black individuals, via the development of culturally tailored community education and outreach programs. The collective engagement of academic institutions, FBOs, CBOs, CommRx, government stakeholders, HCPs, and front-line workers in communities where systemic injustice has historically prevailed was an opportunity to demonstrate that sustainable, authentic, and collaborative CAPP partnerships are integral parts of public health and can be established via intentional, stepwise engagement with underserved communities and trusted community stakeholders [21,29].

## Figures and Tables

**Table 1:** Participant characteristics including social determinants of health and COVID-19 prevention behaviors.

	Total (*n* = 527)	FBO (*n* = 249)	Comm Rx (*n* = 101)	NBHD (*n* = 83)	CBO (*n* = 94)	*p*-Value
	*n* (%)	*n* (%)	*n* (%)	*n* (%)	*n* (%)	
Demographic Characteristics						
Age (years), Mean ± SD	53.3 ± 16.1	55.0 ± 17.1	52.2 ± 16.5	52.7 ± 15.1	50.6 ± 13.4	0.120
Woman	300 (56.9)	148 (59.4)	57 (56.4)	57 (68.7)	38 (40.4)	0.001
Race						<0.001
Black	474 (89.9)	230 (92.4)	91 (90.1)	62 (74.7)	91 (96.8)	
White	24 (4.6)	3 (1.2)	7 (6.9)	14 (16.9)	0 (0.0)	
Other^‡^	22 (4.2)	12 (4.8)	2 (2.0)	5 (6.0)	3 (3.2)	
Unknown	7 (1.3)	4 (1.6)	1 (1.0)	2 (2.4)	0 (0.0)	
Social Determinants of Health (SDoH)						
Educational attainment						<0.001
High school education or less	318 (60.3)	130 (52.2)	70 (69.3)	43 (51.8)	75 (79.8)	
Greater than high school education	203 (38.5)	115 (46.2)	29 (28.7)	40 (48.2)	19 (20.2)	
Unknown	6 (1.1)	4 (1.6)	2 (2.0)	0 (0.0)	0 (0.0)	
Household Income						<0.001
<USD 25,000	256 (48.6)	94 (37.8)	60 (59.4)	33 (39.8)	69 (73.4)	
≥USD 25,000	209 (39.7)	119 (47.8)	32 (31.7)	37 (44.6)	21 (22.3)	
Prefer not to answer/Unknown	62 (11.8)	36 (14.5)	9 (8.9)	13 (15.7)	4 (4.3)	
Employed (Part-time or full-time)	182 (34.5)	101 (40.6)	30 (29.7)	30 (36.1)	21 (22.3)	0.010
Saw a healthcare provider in the past 12 months	463 (87.9)	223 (89.6)	85 (84.2)	67 (80.7)	88 (93.6)	0.031
Low health literacy	75 (14.2)	22 (8.8)	27 (26.7)	8 (9.6)	18 (19.2)	<0.001
Owns insurance	479 (90.9)	222 (89.2)	92 (91.1)	80 (96.4)	85 (90.4)	0.266
COVID-19 Prevention Behaviors, SDoH, and Motives to Vaccinate						
Engaged in preventive behaviors	460 (87.3)	223 (89.6)	92 (91.1)	63 (75.9)	82 (87.2)	0.007
Wore face covering or mask	511 (97.0)	243 (97.6)	99 (98.0)	78 (94.0)	91 (96.8)	0.356
Washed hands with soap	504 (95.6)	243 (97.6)	96 (95.1)	77 (92.8)	88 (93.6)	0.178
Distanced with six feet	484 (91.8)	233 (93.6)	95 (94.1)	70 (84.3)	86 (91.5)	0.048
COVID-19-related SDoH						
Obtaining the healthcare I need	163 (30.9)	79 (31.7)	34 (33.7)	32 (38.6)	18 (19.2)	0.032
Having a place to live	99 (18.8)	40 (16.1)	22 (21.8)	19 (22.9)	18 (19.2)	0.435
Having enough food to eat	137 (26.0)	61 (24.5)	24 (23.8)	29 (34.9)	23 (24.5)	0.249
Having clean water to drink	95 (18.0)	40 (16.1)	20 (19.8)	19 (22.9)	16 (17.0)	0.521
Obtaining the medicine I need	108 (20.5)	50 (20.1)	27 (26.7)	19 (22.9)	12 (12.8)	0.103
Getting to where I need to go	138 (26.2)	70 (28.1)	29 (28.7)	21 (25.3)	18 (19.2)	0.354
Number of COVID-19-related challenges, Mean ± SD	1.4 ± 1.9	1.4 ± 2.0	1.5 ± 2.0	1.7 ± 2.0	1.1 ± 1.7	0.223
Trusted sources of COVID-19 information						
Doctor/HCP	401 (76.1)	185 (74.3)	78 (77.2)	60 (72.3)	78 (83.0)	0.305
Faith leader	302 (57.3)	169 (67.9)	53 (52.5)	27 (32.5)	53 (56.4)	<0.001
Close friends and members of your family	286 (54.3)	131 (52.6)	58 (57.4)	33 (39.8)	64 (68.1)	0.002
The U.S. Coronavirus Task Force	268 (50.9)	139 (55.8)	49 (48.5)	33 (39.8)	47 (50.0)	0.079
City or town government	244 (46.3)	113 (45.4)	50 (49.5)	35 (42.2)	46 (48.9)	0.721
The Louisiana state government	241 (45.7)	113 (45.4)	50 (49.5)	32 (38.6)	46 (48.9)	0.440
News on the radio, TV, online, or in newspapers	191 (36.2)	78 (31.3)	47 (46.5)	22 (26.5)	44 (46.8)	0.002
The U.S. government	172 (32.6)	74 (29.7)	38 (37.6)	22 (26.5)	38 (40.4)	0.106
Coworkers, classmates, or others you know	149 (28.3)	67 (26.9)	41 (40.6)	11 (13.3)	30 (31.9)	0.001
Contacts on social media	95 (18.0)	42 (16.9)	25 (24.8)	8 (9.6)	20 (21.3)	0.047
Motivated by concern for community safety and return to normalcy	3.8 ± 2.5	4.3 ± 2.4	3.7 ± 2.4	4.2 ± 2.4	2.3 ± 2.4	<0.001
Motivated by health concerns/HCP recommendations	0.4 ± 0.7	0.5 ± 0.7	0.4 ± 0.7	0.5 ± 0.8	0.2 ± 0.5	0.0004

CommRx = community pharmacy clients; FBO = faith-based organization members; CBO = community-based organization contacts; NBHD = neighborhood residents. ^‡^ Other includes Asian, American Indian/Alaska Native, Native Hawaiian/Other Pacific Islander, more than one race, and those indicating “other race”.

**Table 2:** Likelihood of vaccination using NPS (*n* = 527).

	Promotors (%)	Detractors (%)	NPS (%)
Full sample	60.3	25.8	34.5
FBO	59.4	25.7	33.7
Comm Rx	65.4	20.8	44.6
NBHD	56.6	28.9	27.7
CBO	60.6	28.7	31.9

**Table 3:** Factors associated with likelihood of COVID-19 vaccination.

	Likelihood of COVID-19 Vaccination (*n* = 527)	
	**OR (95% CI)**	***p*-Value**
Age (10-year increments)	1.47 (1.27, 1.72)	<0.001
CommRx (vs. FBO)	1.93 (1.02, 3.65)	0.044
CBO (vs. FBO)	2.57 (1.37, 4.83)	0.003
Engaged in preventive behaviors	1.88 (0.94, 3.76)	0.075
Motivated by health/doctor recommendation	1.45 (0.93, 2.25)	0.099
Motivated by concern for community safety and return to normalcy	1.52 (1.36, 1.70)	<0.001
Trust government (a great deal)	4.26 (2.44, 7.43)	<0.001
Trust doctors/HCPs (a great deal)	2.83 (1.64, 4.88)	<0.001
Number of COVID-19-related challenges	1.14 (1.00, 1.29)	0.052

## Data Availability

Data are contained within the article.
